# Key epidemiological drivers and impact of interventions in the 2020 SARS-CoV-2 epidemic in England

**DOI:** 10.1126/scitranslmed.abg4262

**Published:** 2021-07-14

**Authors:** Edward S. Knock, Lilith K. Whittles, John A. Lees, Pablo N. Perez-Guzman, Robert Verity, Richard G. FitzJohn, Katy A. M. Gaythorpe, Natsuko Imai, Wes Hinsley, Lucy C. Okell, Alicia Rosello, Nikolas Kantas, Caroline E. Walters, Sangeeta Bhatia, Oliver J. Watson, Charlie Whittaker, Lorenzo Cattarino, Adhiratha Boonyasiri, Bimandra A. Djaafara, Keith Fraser, Han Fu, Haowei Wang, Xiaoyue Xi, Christl A. Donnelly, Elita Jauneikaite, Daniel J. Laydon, Peter J. White, Azra C. Ghani, Neil M. Ferguson, Anne Cori, Marc Baguelin

**Affiliations:** 1 MRC Centre for Global Infectious Disease Analysis, Abdul Latif Jameel Institute for Disease and Emergency Analytics (J-IDEA), School of Public Health, Imperial College London, London W2 1PG, UK.; 2 National Institute for Health Research (NIHR) Health Protection Research Unit (HPRU) in Modelling and Health Economics, London, UK.; 3 Modelling and Economics Unit, National Infection Service, Public Health England, London NW9 5EQ, UK.; 4 Department of Infectious Disease Epidemiology, Faculty of Epidemiology and Population Health, London School of Hygiene and Tropical Medicine, London WC1E 7HT, UK.; 5 Faculty of Natural Sciences, Department of Mathematics, Imperial College London, London SW7 2BX, UK.; 6 Department of Infectious Disease, School of Public Health, Imperial College London, London W2 1PG, UK.; 7 Department of Statistics, University of Oxford, Oxford OX1 3LB, UK.; 8 NIHR HPRU in Emerging and Zoonotic Infections, Liverpool, UK.

## Abstract

We fitted a model of SARS-CoV-2 transmission in care homes and the community to regional surveillance data for England. Compared with other approaches, our model provides a synthesis of multiple surveillance data streams into a single coherent modeling framework, allowing transmission and severity to be disentangled from features of the surveillance system. Of the control measures implemented, only national lockdown brought the reproduction number (*R_t_*^eff^) below 1 consistently; if introduced 1 week earlier, it could have reduced deaths in the first wave from an estimated 48,600 to 25,600 [95% credible interval (CrI): 15,900 to 38,400]. The infection fatality ratio decreased from 1.00% (95% CrI: 0.85 to 1.21%) to 0.79% (95% CrI: 0.63 to 0.99%), suggesting improved clinical care. The infection fatality ratio was higher in the elderly residing in care homes (23.3%, 95% CrI: 14.7 to 35.2%) than those residing in the community (7.9%, 95% CrI: 5.9 to 10.3%). On 2 December 2020, England was still far from herd immunity, with regional cumulative infection incidence between 7.6% (95% CrI: 5.4 to 10.2%) and 22.3% (95% CrI: 19.4 to 25.4%) of the population. Therefore, any vaccination campaign will need to achieve high coverage and a high degree of protection in vaccinated individuals to allow nonpharmaceutical interventions to be lifted without a resurgence of transmission.

## COVID in the community

Understanding factors related to the initial spread and control of SARS-CoV-2 is important in light of emerging variants. Knock *et al*. retrospectively examined differences in SARS-CoV-2 transmission and related mortality in care homes, hospitals, and the community in England since the virus was first introduced in December 2020, stratified by age and geographical region over time. They found that lockdown was by far the most effective control measure and also estimated that mortality in England at this time could have been roughly halved if a lockdown had been introduced 1 week earlier.

## Teaser

A SARS-CoV-2 transmission model fitted to surveillance data estimates virus transmissibility, infection severity, and the impact of interventions.

## INTRODUCTION

England is among the countries worst affected by the global pandemic of coronavirus disease 2019 (COVID-19), caused by the novel *Betacoronavirus* severe acute respiratory syndrome coronavirus 2 (SARS-CoV-2). More than 66,000 deaths are reported to have occurred by 2 December 2020 in England or 117 deaths per 100,000 people (*1*). The impact of the epidemic has varied across the country, with regional epidemics differing in their severity and timing. A key feature in all regions is the burden suffered by older adults living in care homes, where mortality has been high.

We used a mathematical model of SARS-CoV-2 transmission to reproduce the first two waves of the epidemic across England’s seven National Health Service (NHS) regions and assess the impact of interventions implemented by the U.K. government. We analyzed the epidemic from the importation of SARS-CoV-2 into each region in 2 December 2020, encompassing the first national lockdown from March to May 2020, with the interventions implemented as COVID-19 deaths increased again in the autumn, and the second national lockdown in November.

We developed an age-structured stochastic transmission model of SARS-CoV-2, representing care homes, hospital clinical pathways, and the wider community. We used a Bayesian evidence synthesis approach to estimate model parameters and to reconstruct regional epidemics using data from daily recorded deaths, polymerase chain reaction (PCR) testing, hospital admissions, hospital bed occupancy, individual patient outcomes, contact surveys, and serological surveys. This approach, based on integrating multiple data streams into a single coherent modeling framework, ensured robust epidemiological estimates where characteristics of transmission and severity of SARS-CoV-2 could be disentangled from features of the surveillance system. We evaluated temporal changes in transmission as new control measures were implemented and then relaxed, and population immunity accrued. Inclusion of serological data (accounting for seroreversion) allowed us to robustly estimate region- and age-specific disease severity, to compare severity in care home residents to elderly individuals in the community, and to estimate the total epidemic size by calculating the proportion of individuals infected over time in each region. Last, we examined counterfactual epidemic scenarios, varying the date and duration of the first national lockdown and the effectiveness of restricting care home visits, to quantify the resulting impact on mortality.

Our analysis, which integrates multiple data sources and parametrically accounts for their biases, provides a balanced overview of transmission, hospitalization, and mortality patterns of SARS-CoV-2 in the first and second waves (up to 2 December) in all regions of England. Our results provide crucial insights for controlling the epidemic in the future, emphasizing the importance of acting fast to save lives.

## RESULTS

### Epidemic trajectory

**Fig. 1:**
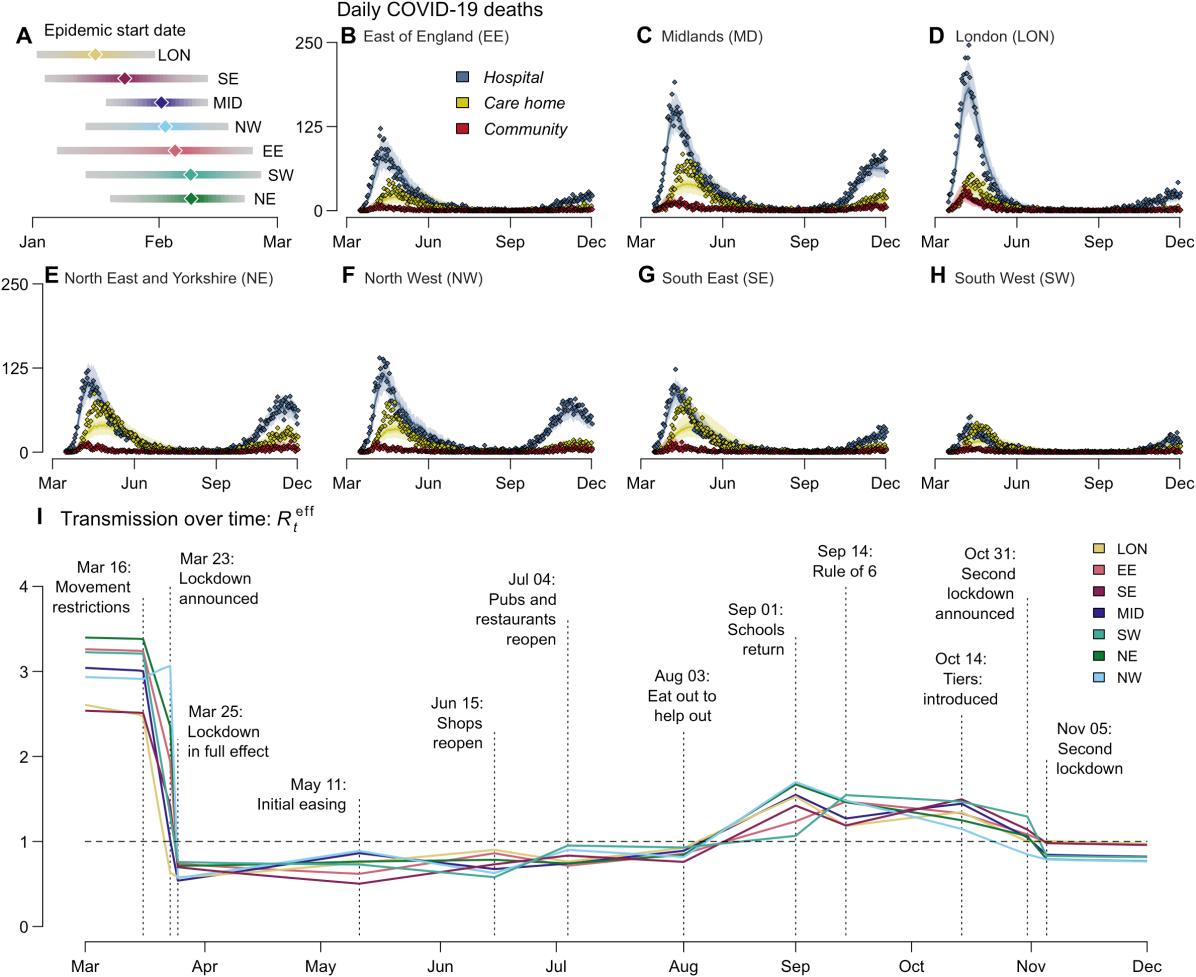
Trajectory of the England COVID-19 epidemic. (**A**) Inferred epidemic start date in each NHS England region. (**B** to **H**) Model fit to reported daily deaths from COVID-19 in care homes, hospitals, and in the community (that is, neither in a hospital nor a care home) for each NHS England region. Points show the daily data (see section S1.1.2 for details of data sources) (*1*). Solid lines show the median posterior, and the shaded area shows the 95% CrI. (**I**) Mean estimated effective reproduction number within the general community (excluding care homes) in each region from March to December 2020. Vertical lines and labels represent dates of key policy changes, defining the breaking points of the underlying piecewise linear transmission rate. Dashed horizontal line depicts a reproduction number (*R*_0_) of 1.

We used estimates of clinical progression to parameterize a stochastic compartmental Susceptible-Exposed-Infectious-Recovered (SEIR)–like transmission model incorporating care homes and hospitalization pathways. Incorporating these estimates into our evidence synthesis approach, we inferred the COVID-19 epidemic start date (assumed as the date when 30 asymptomatic infectious individuals were reached) in each NHS England region (Fig. 1A) and then reconstructed epidemic trajectories for hospitalizations (fig. S1) and deaths in care homes and hospitals (Fig. 1, B to H). Noticeably, throughout England, deaths in care homes peaked, on average, 13 days later than hospital deaths (Fig. 1, B to H).

We estimated the basic reproduction number *R*_0_ (defined as the expected number of onward infections from an infectious individual in a fully susceptible population) to be 2.8 [95% credible interval (CrI): 2.5 to 3.3] nationally. Figure 1I shows how the effective reproduction number *R_t_*^eff^ (the average number of secondary cases generated by an individual infected at time *t*) changed in each region over time in relation to government control measures and accrual of population immunity.

The first COVID-19 death in England occurred on 5 March 2020 (*2*). Seven days later, in response to the growing epidemic, the government began to introduce control measures, initially requiring individuals with a dry persistent cough or fever to self-isolate (*3*). On 23 March, this escalated to a full national lockdown (*3*). Irrespective of initial differences, the degree of transmission during lockdown was similar across all regions (Fig. 1I), consistent with mobility data showing that movement during lockdown reduced to a consistent level nationally (*4*).

We estimated that the epidemic in London and the South East began about 2 weeks before the rest of the country (Fig. 1A), meaning that the lockdown occurred at a later stage of the epidemic in those areas. London experienced an estimated mortality of 96.3 (95% CrI: 84.7 to 108.4) per 100,000 during the first wave, compared with the estimated national average of 86.4 (95% CrI: 75.8 to 99.1), despite having a younger population and a smaller estimated care home population than other regions (294 versus 603 per 100,000 nationally) (*5*).

The first lockdown in England continued until 11 May 2020, when people unable to work remotely were permitted to resume their jobs. Over the summer, restrictions were successively eased, with nonessential shops, pubs, and restaurants opening, followed by the government’s “Eat Out to Help Out” restaurant subsidy scheme in August (*6*). This led to a steady increase in transmission, with *R_t_*^eff^ estimated to rise above 1 in all regions by mid-August (Fig. 1I).

**Fig. 2:**
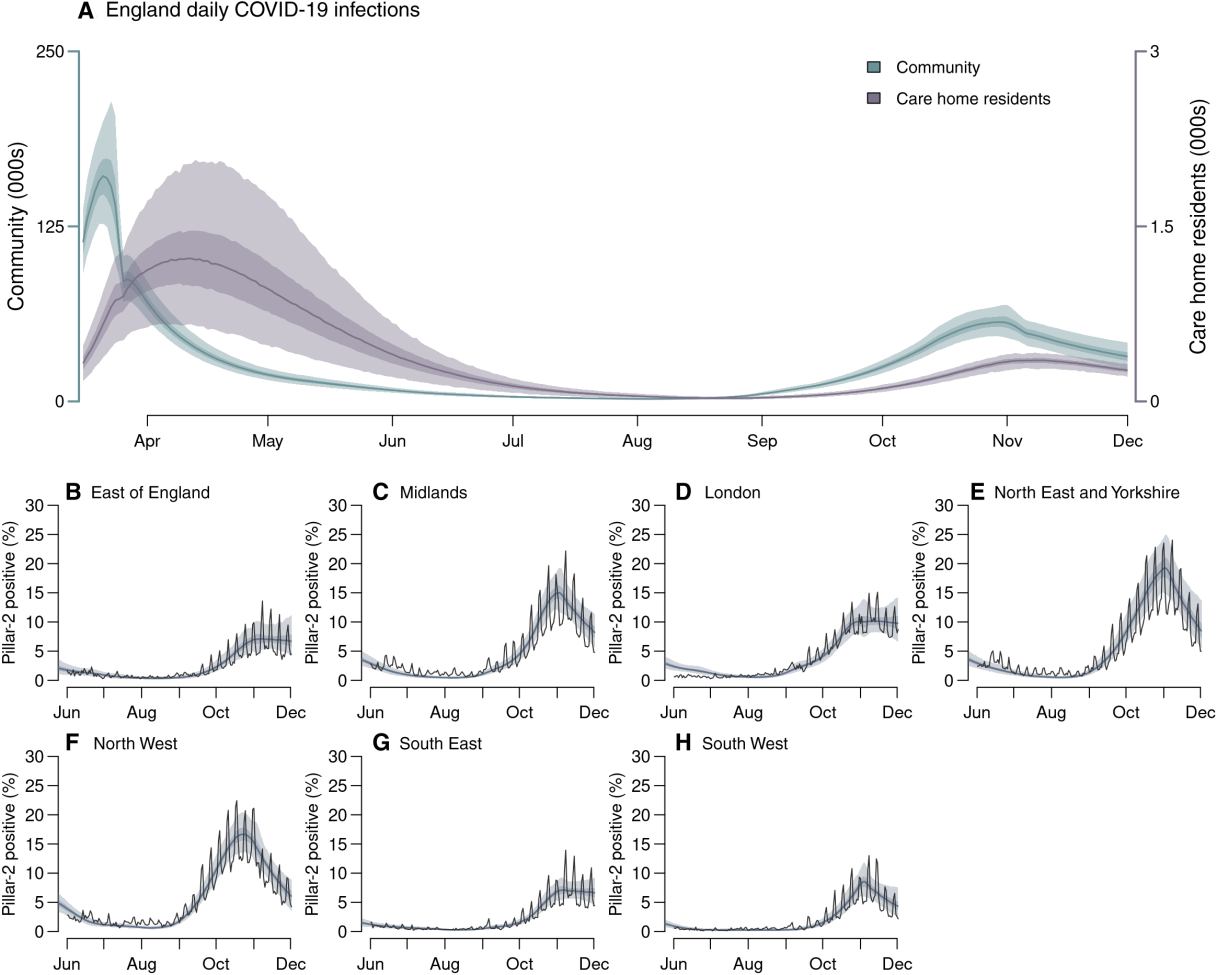
Infection incidence and case positivity over time. (**A**) Inferred daily SARS-CoV-2 infections in England care home residents (excluding care home workers; right axis) and the wider community (left axis). (**B** to **H**) Comparison of modeled (shaded bands) and observed (solid line) proportion of PCR tests that were positive under pillar-2 testing (community swab testing for symptomatic individuals) in >25-year-olds. Shaded bands depict 95% CrI, 50% CrI, and median model outputs.

In common with other European countries, a key feature of the first epidemic wave in England was the high death toll within care homes, which accounted for an estimated 28% of COVID-19 deaths as of 1 August 2020. Although community transmission rates fell during lockdown, our model suggested that transmission within care homes continued to rise, with infection risk peaking in care home residents between 31 March in London and 20 April in the East of England (Fig. 2A).

Increasing PCR test positivity marked the beginning of a second epidemic wave (Fig. 2, B to H, and fig. S6). The accompanying introduction of nonpharmaceutical interventions (NPIs) began with the “rule of six” (limiting social gatherings to six persons maximum) on 14 September (*7*), followed by the localized tiered restrictions on 14 October (*8*). These measures limited transmission in most regions, but our model suggests that they were insufficient to reduce *R_t_*^eff^ below 1 (Fig. 1I). Consequently, on 31 October, the government announced a second national lockdown, which lasted from 5 November to 1 December (*9*).

Restrictions during the second lockdown were less stringent than the first, with schools and some workplaces remaining open. This was reflected in *R_t_*^eff^ estimates of 0.88 (95% CrI: 0.82 to 0.95) on 18 November 2020, the midpoint of the second lockdown, compared to *R_t_*^eff^ *=* 0.68 (95% CrI: 0.65 to 0.72) on 16 April 2020, the midpoint of the first lockdown. We estimated that, without the population immunity accrued during the first wave, contact rates during the second lockdown would have resulted in a reproduction number of *R_t_ =* 1.05 (95% CrI: 0.97 to 1.14). Hence, population immunity helped to reduce transmission below the critical threshold of *R_t_*^eff^ = 1.

### Severity and hospitalization

**Fig. 3:**
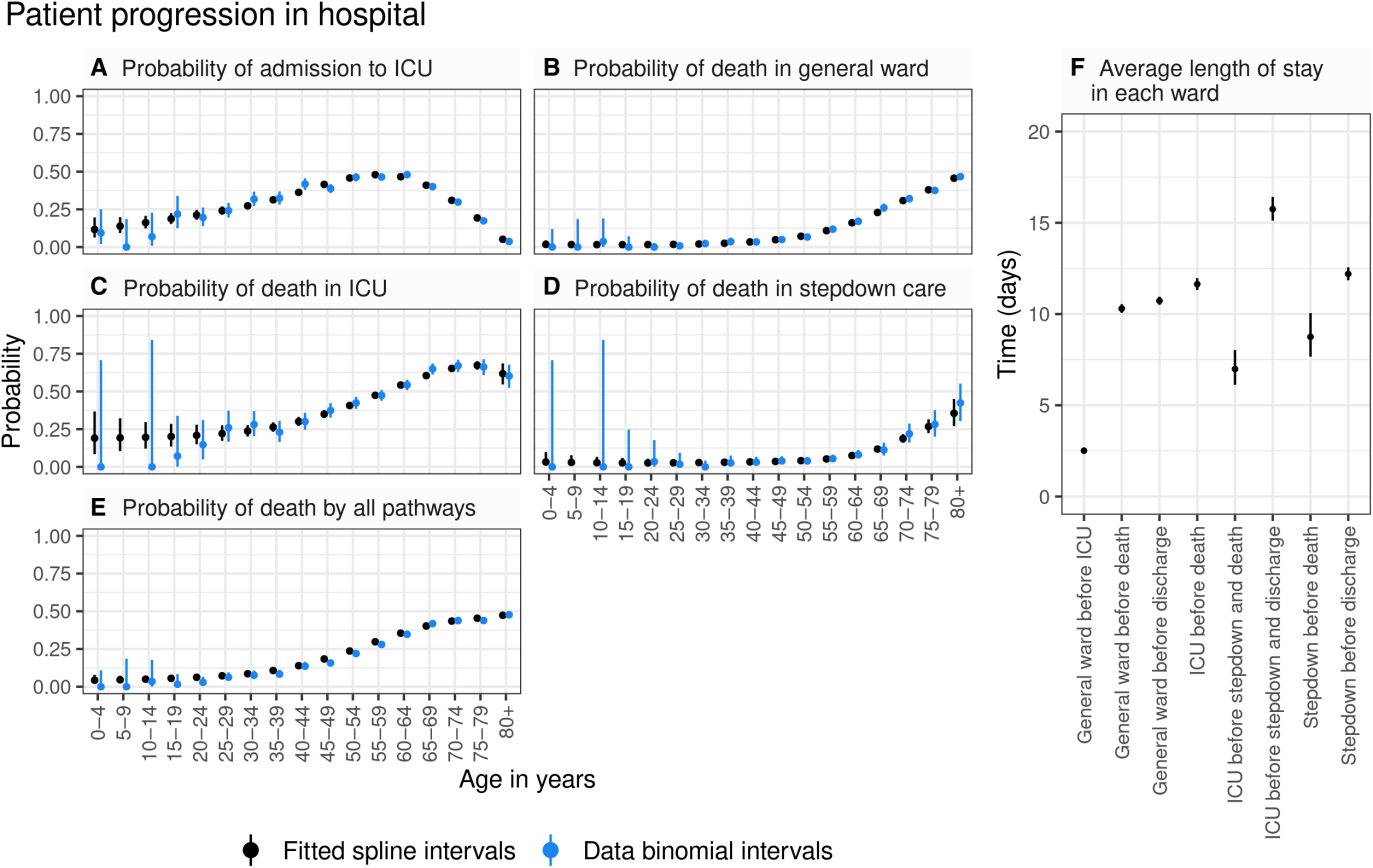
Age-dependent probabilities of progression through hospital pathways. (**A**) Probability of admission to ICU. (**B**) Probability of death in a general hospital ward. (**C**) Probability of death in an ICU. (**D**) Probability of death in hospital during stepdown care. (**E**) Probability of death through all hospital pathways [obtained by combining (B) to (D) using the branching structure shown in fig. S4]. Black circles and vertical segments show the posterior mean and 95% CrI of splines fitted to data, and blue circles and vertical segments show raw data mean values and 95% confidence intervals (exact binomial) for each 5-year age group. (**F**) Average estimated length of stay in each ward (posterior mean and 95% CrI).

COVID-19 manifests a broad spectrum of severity, from asymptomatic infection to life-threatening illness requiring intensive care. We estimated age patterns of clinical progression in people admitted to hospital using individual-level data from 17,702 patients admitted between 18 March and 31 May 2020 (inclusive) in the COVID-19 Hospitalisation in England Surveillance System [CHESS; (*10*)]. We derived estimates of the time spent in each stage of the hospital pathway [including general wards, intensive care unit (ICU), and post-ICU stepdown care], as well as age-stratified probabilities of progression through that pathway (Fig. 3, A to E, and fig. S2). Accounting for differing lengths of stays given different outcomes, there were marked differences in the average length of ICU stay for those who died in the ICU, those who later died in stepdown care, and those who were discharged after stepdown care (Fig. 3F). Among patients over 65 years of age, we found that the probability of admission to ICU decreased with increasing age. Thus, it is possible that older and more severely infected patients were directed to care on a general ward rather than admitted to ICU where the benefits of ventilation and the corresponding prognosis may not be better than with oxygen therapy in a general ward (*11*).

**Fig. 4:**
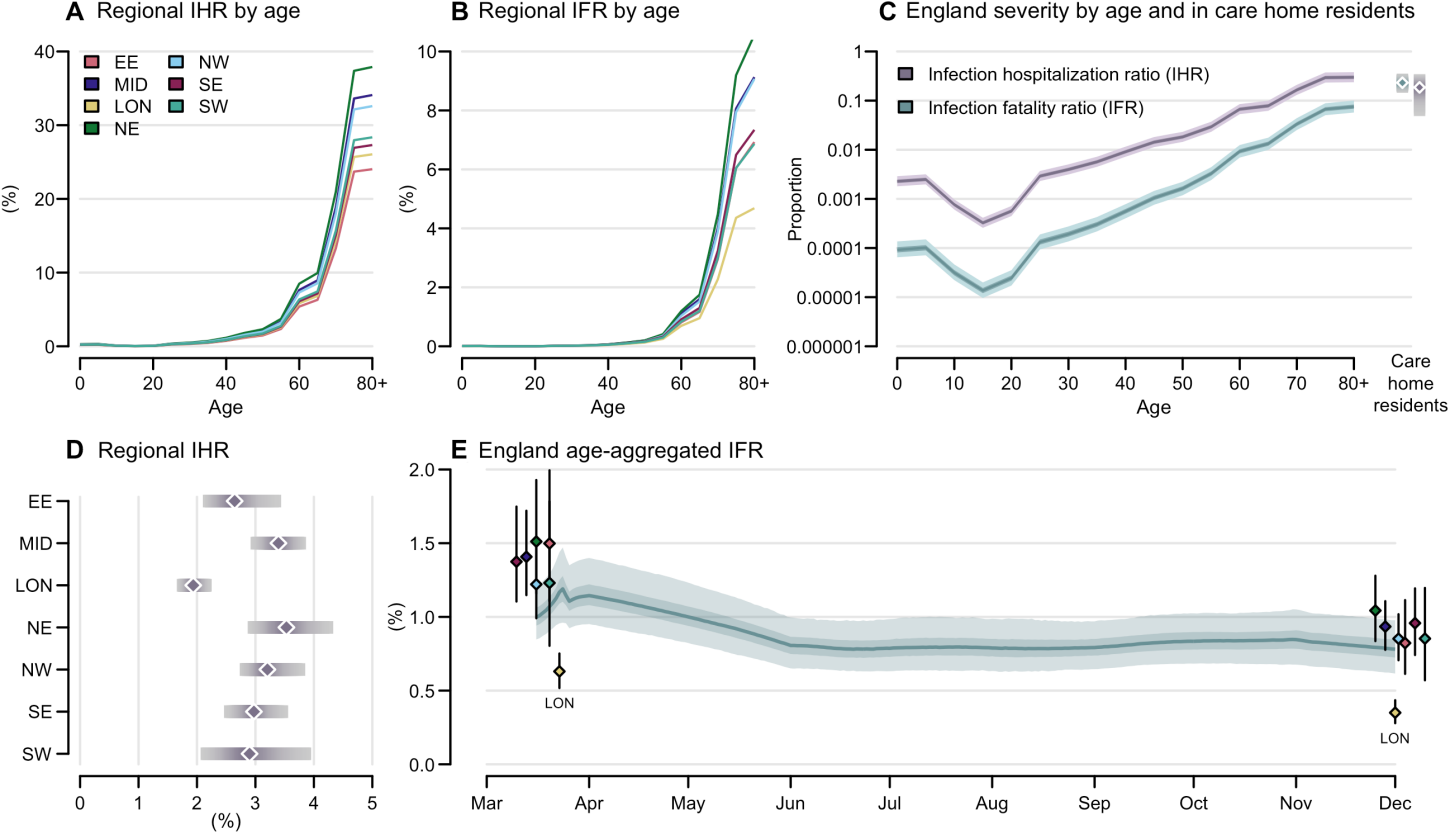
Estimated relative severity of disease by age group and region. (**A** and **B**) Variation in (A) the median inferred infection fatality ratio (IFR) and (B) infection hospitalization ratio (IHR) by age group in each region. Ages 80+ were modeled as a single risk group; care home residents were not included. (**C**) Estimated England IFR and IHR by age group and in care home residents (estimate excludes care home workers). National severity estimates are produced by aggregating regional estimates on the basis of infection incidence. (**D**) Regional estimated IHR, aggregated over age and risk group by infection incidence. Plots in (A) to (D) use parameter estimates and incidence weightings calculated as of 1 December 2020. (**E**) Estimated England IFR over time; colored dots show regional estimates of IFR at the start of the epidemic and on 1 December 2020 [clusters each correspond to one time point, London (LON)]. In (C) to (E), shaded bands depict 95% CrI and interquartile ranges, and points depict medians.

We used estimates of clinical progression to parameterize the transmission model, enabling us to infer temporal and regional differences in disease severity informed by local demography, observed daily hospital admissions, bed occupancy, and deaths. We measured the severity of disease by the infection fatality ratio (IFR) and the infection hospitalization ratio (IHR). The severity of disease increased with age in all regions with the steepest increase above 65 years (Fig. 4, A to C), in line with observations worldwide (*5*). Regional estimates of age-aggregated disease severity depended on the population age distribution, which was similar in most regions of the country, except London, where the median age was 34.6 years (versus 39.5 years nationally). At the start of the first wave, London experienced an estimated IFR of 0.63% (95% CrI: 0.52 to 0.75%) compared to the estimated national average of 1.00% (95% CrI: 0.85 to 1.21%). The IHR in London was 1.94% (95% CrI: 1.68 to 2.25%) compared to the national average of 2.55% (95% CrI: 2.17 to 3.04%) (Fig. 4, D and E). Regional variation in the population age distribution did not fully account for differences in severity, with London still experiencing lower mortality when stratified by age (Fig. 4, A and B). The oldest age group (80+) in London had an estimated IFR of 4.7% (95% CrI: 3.6 to 6.2%) compared to 10.7% (95% CrI: 8.2 to 13.8%) in the North East and Yorkshire.

We estimated temporal trends in the overall IFR for England by weighting regional estimates by incidence. At the start of the first wave, the estimated national IFR was 1.00% (95% CrI: 0.85 to 1.21%) (Fig. 4E), consistent with earlier reports from serology data alone (*12*). The national IFR initially appeared to increase as transmission widened from London to regions with older populations and greater disease severity. Over the first wave, the proportion of hospital admissions resulting in death decreased, likely due to improvements in clinical management and alleviation of capacity constraints (*13*), leading to an estimated national IFR of 0.79% (95% CrI: 0.63 to 0.99%) by the end of the first wave. The magnitude of the relative reduction in IFR over time varied between regions, from an estimated 29.8% (95% CrI: 15.5 to 42.4%) in the North West to 44.6% (95% CrI: 28.4 to 57.7%) in East of England.

The inferred IFR was greater among care home residents (23.3%, 95% CrI: 14.7 to 35.2%) than in the 80+ in the community (7.9%, 95% CrI: 5.9 to 10.3%; Fig. 4C). Many care home residents did not transfer into hospital and instead died in the facilities where they lived; so conversely, the inferred IHR was lower in care home residents (18.8%, 95% CrI: 4.9 to 34.6%) than in those aged 80+ in the community (30.7%, 95% CrI: 24.1 to 38.9%). We present national estimates of severity at the end of the second wave, stratified by age and care home residency, in table S9.

### Epidemic size

**Fig. 5:**
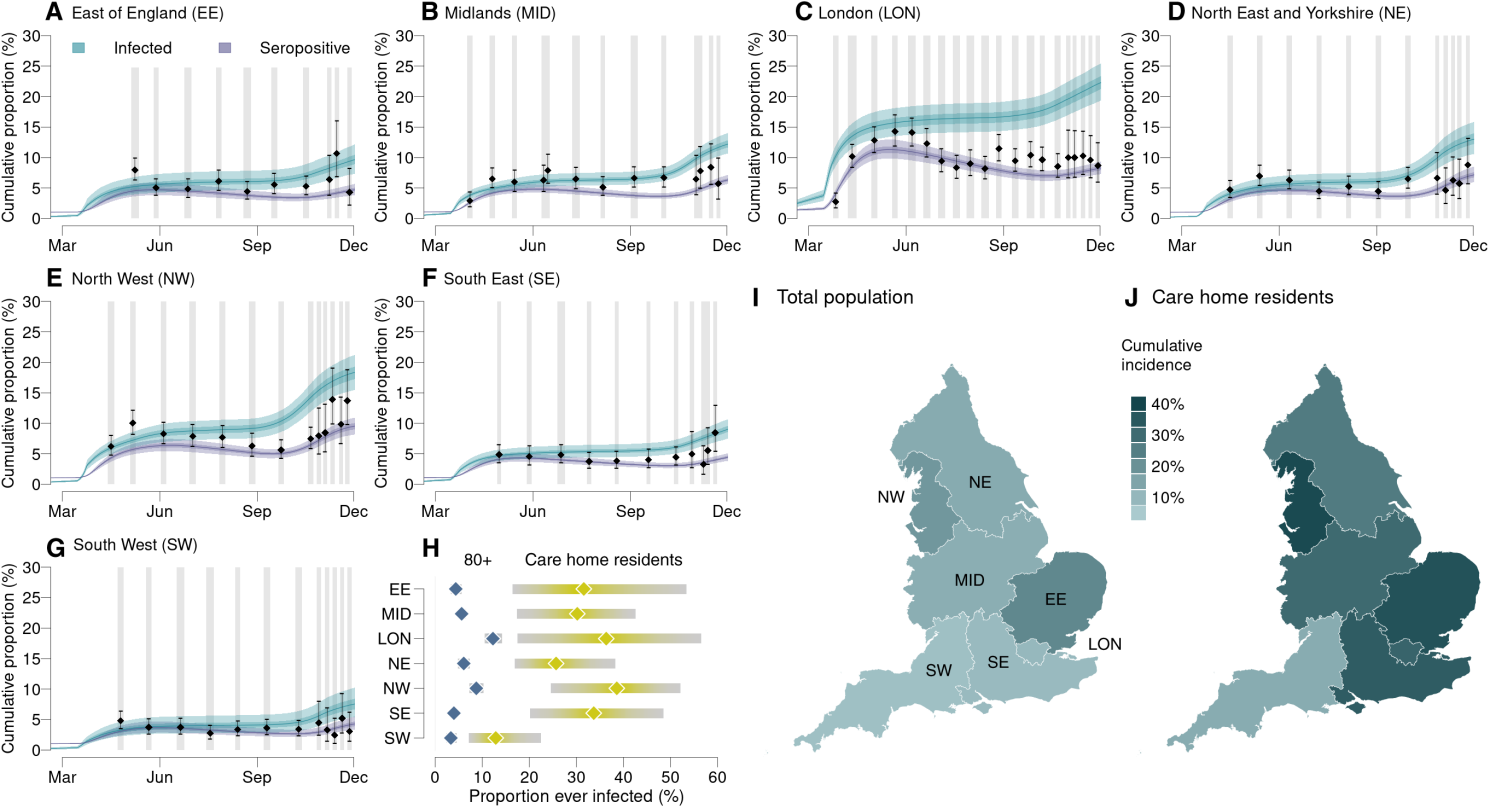
Cumulative COVID-19 incidence and seropositivity by region. (**A** to **G**) Comparison of the estimated proportion of the population testing seropositive in 2020 with observations from serological surveys [see section S1.1.4 (*14*)]. Vertical gray shaded bands show serological survey timings, black points show the observed seroprevalence (bars, 95% exact confidence intervals), and blue and purple lines show the proportion of the population infected and seropositive, respectively, as inferred from our model (shaded bands show the 95% CrI, 50% CrI, and median). (**H**) Comparison by region of the estimated cumulative attack rate (median and 95% CrI) in care home residents (yellow, excludes care home workers) versus in the 80+ age group in the community (blue). The estimated mean final epidemic size in each England NHS region (**I**) in total and (**J**) in care home residents (excludes care home workers).

Data from repeated serological surveys of blood donors aged 17 to 65 informed our estimation of the total regional 2020 epidemic size (Fig. 5, A to G), accounting for the imperfect sensitivity and specificity of serological tests alongside seroreversion (*14*). Seropositivity notably declined after the first wave in some regions (Fig. 5, A to G). This reflected not only seroreversion (*15*) but also likely temporal trends in the composition of the surveyed population. Lockdown restrictions made attending blood donation centers difficult for all except key workers, who were more likely to have been infected (*16*) and may therefore be overrepresented in the sample of blood donors during the two lockdowns. The decline of seropositivity is modeled independently of population immunity, which was assumed not to wane over the study period.

The estimated cumulative proportion of the population ever infected with SARS-CoV-2 ranged from 7.6% (95% CrI: 5.4 to 10.2%) in the South West to 22.3% (95% CrI: 19.4 to 25.4%) in London (Fig. 5H). The increase in seropositivity lagged cumulative infections by 2 weeks, reflecting the time from infection to seroconversion.

The estimated proportion of care home residents ever infected with SARS-CoV-2 was 29.8% (95% CrI: 17.6 to 44.0%), much higher than the 6.1% (95% CrI: 5.0 to 7.2%) estimated in >80-year-olds living in the community. This difference was consistent across most regions (Fig. 5H) where regional differences in the estimated care home attack rates mirrored the patterns estimated in the general community, with regions with larger community epidemics also experiencing larger care home epidemics (Fig. 5, I and J). In the East of England and the South East, this pattern was decoupled, likely due to slight inconsistencies in how COVID-19 cases were reported between different datasets.

### Impact of NPIs

**Fig. 6:**
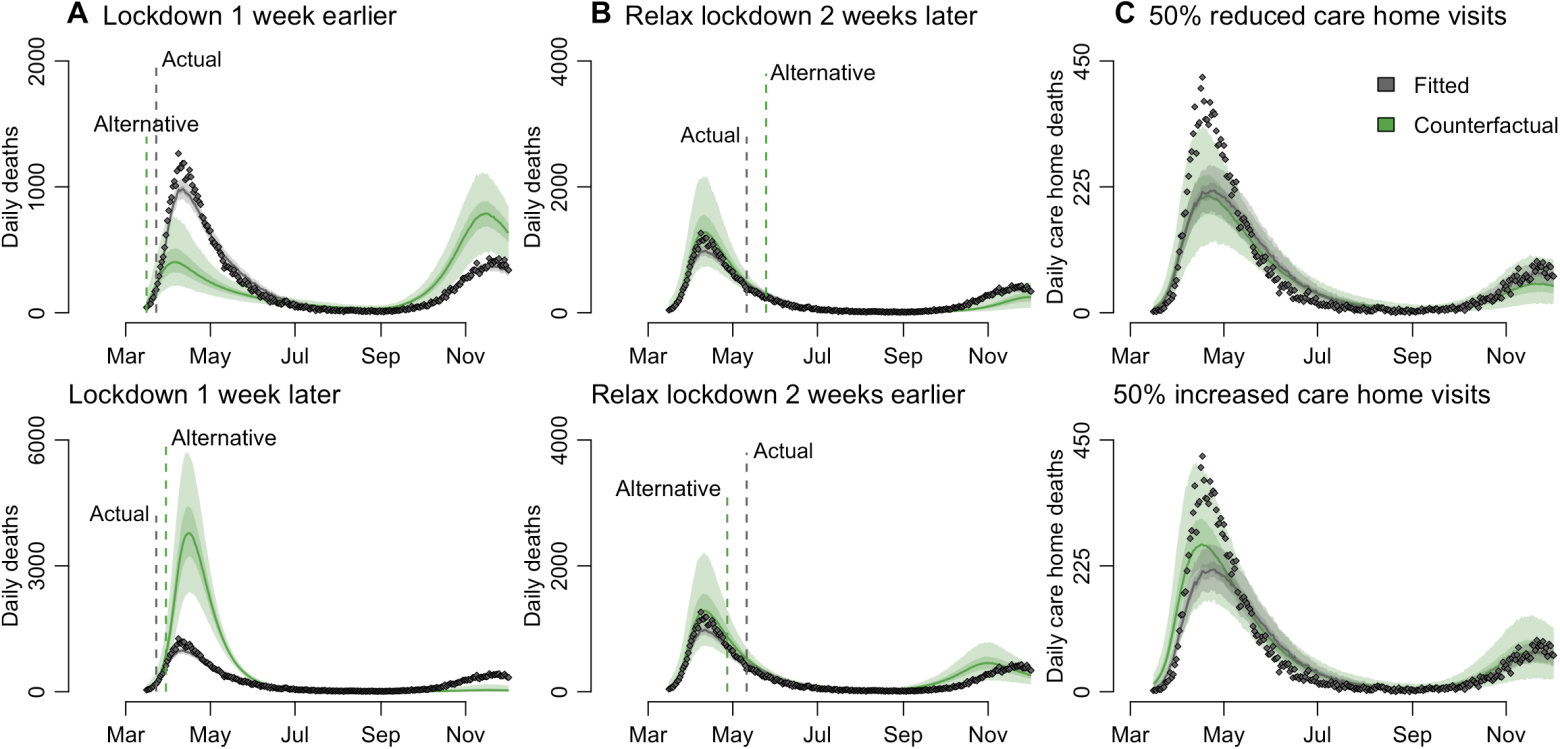
Counterfactual analysis of the impact on mortality aggregated across NHS England regions. We estimated the impact of (**A**) initiating lockdown 1 week earlier/later, (**B**) relaxing lockdown 2 weeks earlier/later, and (**C**) in response to 50% more/less restricted care home visits from March to November. (A) and (B) present counterfactual outcomes for daily deaths in England but have different *y*-axis scales to better highlight differences between the observed data and each alternative lockdown scenario. In all panels, gray dots depict data [see section S1.1.2 for details of data sources (*1*)]. Gray and green solid lines show the posterior median for the fitted and counterfactual model, respectively, and shaded bands depict the corresponding 95% CrI and interquartile ranges. Vertical dashed lines indicate the timings of the actual and alternative (used in the counterfactual analysis) interventions, respectively. Figure S3 presents a regional breakdown of this figure.

We explored counterfactual intervention scenarios and examined the potential impact on mortality of initiating the first national lockdown 1 week earlier or later, ending that lockdown 2 weeks earlier or later, and with 50% more or less restricted care home visits throughout the epidemic (Fig. 6). We found that the timing of the initial national lockdown was crucial in determining the eventual epidemic size in England. We estimated that locking down a week earlier could have reduced the first wave death toll (up to 1 July 2020) from an estimated 48,600 to 25,600 (95% CrI: 15,900 to 38,400), whereas delaying lockdown by a week would have increased the number of deaths to 132,800 (95% CrI: 91,900 to 180,700) (Fig. 6A). Note that an earlier lockdown could also result in a larger second wave, which could then be avoided by moving autumn control measures earlier. The estimated impact of such an approach varied by region, with regions with less established epidemics at the time of the first lockdown more sensitive to the timing of the intervention (fig. S3, A and B). Conversely, we estimated that locking down a week later may have increased deaths, with large variability by region, from 117% in London to 248% in the North East and Yorkshire but with very large uncertainty (fig. S3B). Initiating a lockdown to interrupt the exponential growth phase of an epidemic has a much greater impact on reducing total mortality than extending an existing lockdown. Because of this asymmetry, we estimated that relaxing the lockdown measures 2 weeks earlier could have increased deaths by 9400 (95% CrI: −6300 to 26,800) before 2 December. Conversely, relaxing measures later could have prevented 8300 (95% CrI: 1100 to 14,100) deaths before 2 December (Fig. 6B).

We also explored counterfactual scenarios varying the extent of visit restrictions in care homes and estimated that reducing contact between the general population and care home residents by 50% would not have markedly affected care home deaths. The fits to care home deaths have lower and slightly later peaks compared with the data (Fig. 6C). This may be due to the different transmission dynamics in care homes at this time of the epidemics with, for example, some NHS patients being discharged to care homes without prior testing. We estimated that deaths could have decreased by up to 30% or increased by up to 24% compared with the median fitted simulations (Fig. 6C).

## DISCUSSION

We present a detailed overview of SARS-CoV-2 transmission, hospitalization, mortality, and intervention impact in the first two epidemic waves across all regions of England between March and December 2020. We successfully reproduced the transmission dynamics of the two epidemic waves, in terms of cases, PCR prevalence, seroprevalence, hospitalized cases (general wards and ICU), and deaths in hospitals and in care homes.

We estimated that intense transmission was occurring in care homes even during the first national lockdown when *R_t_*^eff^ in the community was well below 1 in all regions (*17*–*19*). Combined with our counterfactual analysis of restricting visits, this suggests that reducing infections in care home residents is challenging. This highlights the difficulty of protecting care home residents from COVID-19: Because of the necessarily close contact between staff and residents within a care home, once an outbreak has begun, it is very difficult to reduce transmission, which overrides any impact of reducing the number of introductions (*20*, *21*). The disproportionately high burden of COVID-19 mortality in care homes has been observed in many high-income countries (*22*, *23*), with an estimated IFR between 20 and 40% among care home residents in France assuming that individuals are 3.8 to 6.0 times more frail than the general population (*24*). Our results about transmission in care homes are mitigated by the difficulty of reproducing the dynamics of mortality in care homes in some regions where deaths were underestimated by the model. This might be due to the simple approach used to model care homes and the uncertainty in the actual number of care home deaths as shown in the discrepancy between confirmed deaths and deaths attributed to COVID-19.

We found that, consistent with existing literature both in the United Kingdom (*25*) and globally (*24*), disease severity increased markedly with age. Assessment of severity is complicated by the broad clinical spectrum of COVID-19 (*26*–*28*), the population age structure, and surveillance systems (*29*, *30*). Here, we provide updated severity estimates for England on the basis of multiple contemporary data streams. We estimated considerable regional heterogeneity in infection severity, broadly consistent in the general population and in care homes for IFR and IHR. London experienced the lowest severity even after adjusting for its younger population. The estimated twofold reduction over time in IFR cannot be explained solely by the introduction of dexamethasone, which reduces mortality among ICU patients (*31*), but rather a combination of factors including improvements in clinical management, greater experience in treating patients in ICU, and alleviation of capacity constraints (*13*, *32*).

Our analysis showed large regional variation in burden, especially in the first wave. This is likely due to the pattern of seeding and the timing of national lockdown relative to how advanced each region’s epidemic was. Our counterfactual scenarios of initiating the first national lockdown 1 week earlier or later highlight the importance of early interventions to reduce overall mortality.

Studies of COVID-19 interventions have found that the effectiveness of NPIs depends critically on the local context and when restrictions are implemented relative to how large the epidemic has grown. Across multiple countries, a combination of NPIs was necessary to limit SARS-CoV-2 transmission, with studies finding curfews, lockdowns, and restricting social gatherings (*33*, *34*) or school closures and limits on internal movement being the most effective in reducing transmission (*35*). Our finding that only national lockdown measures consistently reduced the *R_t_*^eff^ below 1 is in agreement with other U.K.-based studies (*36*, *37*).

At the midpoint of the second lockdown, we estimated a higher *R_t_*^eff^ of 0.88 (95% CrI: 0.82 to 0.95) compared to the midpoint of the first lockdown in April 2020 (*R_t_*^eff^ *=* 0.68, 95% CrI: 0.65 to 0.72). Less stringent restrictions were in place in England at this time with schools remaining open. Another study also estimated a smaller impact on transmissibility during the second national lockdown in England compared to during the “circuit breaker” implemented in Wales, which coincided with school half-term holidays (*37*). In addition, the emergence of the B.1.1.7 lineage, which has an estimated 43 to 100% multiplicative transmission advantage, coinciding with the second lockdown may have contributed to the higher *R_t_*^eff^ at this time (*38*, *39*).

Even assuming that immunity did not wane during the first year of the epidemic, our estimates of cumulative incidence over time strongly support the hypothesis that the epidemic decline after the first national lockdown was due to NPIs, with immunity playing a minimal role (*40*). Population-level immunity was insufficient to prevent a second wave of infection in any region, illustrated by the increase in reported cases and deaths that prompted the second national lockdown (*41*). Considerable uncertainties remain about the duration of immunity. For example, the extent and duration of infection-induced immunity to SARS-CoV-2 and its relationship to seropositivity remain unclear. Related seasonal coronaviruses induce immunity that wanes in 1 or 2 years (*42*), although antibody titers after SARS-CoV-1 infection appear to decay more slowly (*43*). Although including such immunity would not affect our results, this renders long-term predictions about the dynamics of SARS-CoV-2 challenging.

With the authorization of the first SARS-CoV-2 vaccines in December 2020, we entered a new phase in the control of the COVID-19 pandemic. However, our estimates of population immunity in 2 December 2020 were low, with regional cumulative attack rates ranging from 7.9 to 22.5%; therefore, any vaccination campaign will need to achieve high coverage and a high degree of protection in vaccinated individuals to allow NPIs to be lifted without a resurgence of transmission. Although vaccinating the most vulnerable age and risk groups will considerably reduce the burden of COVID-19, a large proportion of younger age groups may also need to be vaccinated to reach the immunity threshold for control. Our high estimates of transmission in care homes imply the need for high vaccine uptake there.

Our work has a number of limitations due to simplifying assumptions in our analysis. First, because of the compartmental nature of the model, we did not explicitly model individual care homes, rather the regional care home sector as a whole. However, because care home workers may work across multiple facilities leading to within and between care home transmission, we do not expect the simplification to substantially affect our conclusions, but it might have contributed to the difficulty to reproduce the peak of transmission in care homes. Similarly, we did not model individual households or transmission within and between them. When assessing the impact of NPIs on transmission, we therefore captured population averages rather than the contribution of household and nonhousehold contacts. Second, hospital-acquired infections may have contributed to overall transmission, especially around the peak of the epidemic, and to persistence of infection in England over the summer months (*44*, *45*). Our model does not explicitly represent nosocomial transmission; therefore, such effects will be encompassed within our regional *R_t_*^eff^ estimates. Third, each data stream was subject to competing biases, which we statistically accounted for as far as possible (section S1.1). A key strength of our evidence synthesis approach is that we do not rely on any single data source, combining multiple perspectives to provide a robust overall picture of the epidemic. We model the epidemics in each NHS region in England independently without accounting for transmission between regions; however, most of the movement will be within rather than between regions. Last, limitations of the data meant that we could not consider spatial heterogeneity within regions.

Our analysis provides a detailed overview of transmission, hospitalization, and mortality patterns of COVID-19 in the first and second waves of the epidemic in all regions of England, one of the European countries worst affected by the pandemic. Integration of multiple data streams into a single coherent modeling framework enables us to disentangle transmission and severity from features of the surveillance system, provides robust estimates of the epidemiological characteristics of the COVID-19 epidemic in England, and paves the way toward better understanding the contribution of individual surveillance data streams to the assessment of policy questions. As nationwide vaccination programs are rolled out, our results will help to inform how NPIs are applied in the future.

## MATERIALS AND METHODS

### Study design

We developed a stochastic SEIR-like age-structured compartmental model of the transmission of SARS-CoV-2 in community and care homes in England 2020 with a detailed description of progression into hospital pathways after severe disease (see diagram in fig. S4). Model parameters were fitted to epidemiological data, including hospital admissions and bed occupancy, ICU prevalence, deaths in the community/hospitals/care homes, pillar-2 PCR testing data, REal-time Assessment of Community Transmission (REACT) community surveys, and blood donor serological data (see the graph of the functional relationships linking model outputs, data streams, and parameters in fig. S5). Parameters of the model were estimated, and the posterior distributions of the inferred model parameters were used to compute the epidemiological outcomes relevant to the analysis and to run the counterfactual scenarios.

### Statistical analysis

We first analyzed a line list of 17,702 patients requiring hospitalization from the CHESS. Using a progression model fitted with Markov chain Monte Carlo (MCMC), we derived age-stratified estimates of hospital progression parameters (see details in the Supplementary Materials and data files support_progression.csv and support_severity.csv). These parameter estimates were then used as priors in the larger compartmental transmission model to infer population-level estimates of severity alongside the rest of the parameters from the model. Bayesian estimation of the parameters of our models was performed using particle MCMC for each of the seven NHS regions independently (more details are provided in the Supplementary Materials).

Effective reproduction numbers were estimated from the eigenvalues of the next-generation matrix derived from the posterior distributions of the estimated model parameters. An additional analysis using the EpiEstim R package (*46*) was carried out to test the robustness of this approach (see the Supplementary Materials).

## SUPPLEMENTARY MATERIALS

stm.sciencemag.org/cgi/content/full/13/602/eabg4262/DC1
Materials and Methods Figs. S1 to S16 Tables S1 to S9 Data files S1 to S4 References (*47*–*85*)

## Appendix

View/request a protocol for this paper from *Bio-protocol*.
